# ILMCNet: A Deep Neural Network Model That Uses PLM to Process Features and Employs CRF to Predict Protein Secondary Structure

**DOI:** 10.3390/genes15101350

**Published:** 2024-10-21

**Authors:** Benzhi Dong, Hui Su, Dali Xu, Chang Hou, Zheng Liu, Na Niu, Guohua Wang

**Affiliations:** College of Computer and Control Engineering, Northeast Forestry University, Harbin 150040, China; nefudbz@nefu.edu.cn (B.D.); suhui@nefu.edu.cn (H.S.); nefuxdl@nefu.edu.cn (D.X.); houchang@nefu.edu.cn (C.H.); zhengliu@nefu.edu.cn (Z.L.); niuna_niuniu@nefu.edu.cn (N.N.)

**Keywords:** protein secondary structure, unsupervised protein language model, hybrid neural network architecture, conditional random field

## Abstract

Background: Protein secondary structure prediction (PSSP) is a critical task in computational biology, pivotal for understanding protein function and advancing medical diagnostics. Recently, approaches that integrate multiple amino acid sequence features have gained significant attention in PSSP research. Objectives: We aim to automatically extract additional features represented by evolutionary information from a large number of sequences while simultaneously incorporating positional information for more comprehensive sequence features. Additionally, we consider the interdependence between secondary structures during the prediction stage. Methods: To this end, we propose a deep neural network model, ILMCNet, which utilizes a language model and Conditional Random Field (CRF). Protein language models (PLMs) pre-trained on sequences from multiple large databases can provide sequence features that incorporate evolutionary information. ILMCNet uses positional encoding to ensure that the input features include positional information. To better utilize these features, we propose a hybrid network architecture that employs a Transformer Encoder to enhance features and integrates a feature extraction module combining a Convolutional Neural Network (CNN) with a Bidirectional Long Short-Term Memory Network (BiLSTM). This design enables deep extraction of localized features while capturing global bidirectional information. In the prediction stage, ILMCNet employs CRF to capture the interdependencies between secondary structures. Results: Experimental results on benchmark datasets such as CB513, TS115, NEW364, CASP11, and CASP12 demonstrate that the prediction performance of our method surpasses that of comparable approaches. Conclusions: This study proposes a new approach to PSSP research and is expected to play an important role in other protein-related research fields, such as protein tertiary structure prediction.

## 1. Introduction

The structure of proteins, as the main performers of the various functions of organisms, determines their function and stability [1]. Protein secondary structure is a localized conformation formed within a polypeptide chain by hydrogen bonding among atoms in the backbone. It serves as a crucial link between primary and tertiary structures and is an essential part of understanding the relationship between protein structure and function [2]. The protein secondary structure prediction (PSSP) task aims to characterize the local conformation of proteins by assigning various secondary structure labels. Protein secondary structures are usually categorized into eight types based on different hydrogen bonding modes, namely H (α-helix), T (β-turn), S (Bend), I (π helix), G ($3_{10}$-helix), E (β-strand), B (β-bridge), and C (Other), i.e., 8-state labels. In addition, for convenience of testing and description, these eight types can be categorized into three basic categories, H (Helix), E (Sheet), and C (Coil), i.e., 3-state labels [3]. In recent years, research on PSSP has primarily focused on two directions: first, the fusion of multiple amino acid sequence features to assist in prediction, such as the incorporation of additional evolutionary information from Multiple Sequence Alignment (MSA) [4], Position-Specific Scoring Matrix (PSSM) [5], and Hidden Markov Models (HMMs) [6]; and second, the optimization and integration of different deep learning architectures to enhance the accuracy of predicting the correspondingly secondary structure categories for each amino acid residue in the sequence. However, there remains potential for further improvement in both directions.

Since proteins are homologous, prediction approaches that combine evolutionary information can leverage the structural similarity among homologous proteins, significantly enhancing the accuracy of protein structure prediction. Examples of such approaches include AlphaFold [7], SSpro [8], Porter 5.0 [9], MUFOLD-SS [10], and DNSS2 [11]. However, achieving better results is challenging if only the amino acid sequence is considered while completely disregarding evolutionary information. To address this issue, many approaches attempt to leverage pre-trained language models to extract evolutionary information from amino acid sequences. DeepSeqVec [12] was the first to utilize the natural language model ELMo [13] to assist in prediction; however, this direct adaptation of natural language models for structural prediction did not effectively capture protein features. Subsequent releases of protein language models (PLMs), such as ESM [14], Progen [15], and ProtGTP2 [16], were trained on large amounts of unlabeled data and subsequently fine-tuned for various protein downstream tasks. For example, NetSurfP-3.0 [17] utilizes ESM-1b [14] to obtain feature embeddings for each amino acid sequence and constructs neural network models for prediction. Elnaggar et al. [18] pre-trained and proposed the ProtTrans model on the UniRef50, UniRef100 [19], and BFD [20] datasets, which achieved the best performance in protein structure prediction tasks. The essence of the ProtTrans model is to predict masked amino acid residues (tokens) in the target amino acid sequence through unsupervised learning, ultimately generating protein embeddings that encompass both evolutionary information and sequence features. However, the amino acid position information captured in this manner is not sufficiently comprehensive. Therefore, a position coding approach was introduced to obtain more detailed amino acid sequence features during feature processing. The fusion of these two feature processing methods allows the model to fully capture amino acid sequence feature information when only a single amino acid sequence is used as input, ultimately enhancing prediction accuracy.

Additionally, many approaches aimed at developing lighter and more efficient predictive models by improving and fusing multiple deep learning models, subsequently assigning the corresponding three- or eight-state secondary structure labels through the Softmax layer. However, the outputs of the Softmax layer are independent of one another. Conditional Random Field (CRF) [21], a widely used statistical model for sequence data annotation, offers an effective solution to the aforementioned problem by capturing the complex relationships between protein secondary structure labels through feature functions. The features used in CRF not only encompass the attributes of each amino acid in the sequence but also include interactions between amino acids. Compared to traditional HMMs, CRF utilizes the Viterbi algorithm [22] during the decoding process, which accounts for the entire amino acid sequence through global normalization, thereby enabling accurate identification of the most probable secondary structure tag sequences.

The contributions of this work are twofold:

- We combine two feature processing approaches to obtain rich and comprehensive amino acid sequence representations. First, we utilize feature representations embedded with evolutionary information generated by the PLM, addressing the limitations in acquiring evolutionary information features and the potential for poor predictions if such information is discarded entirely. Additionally, amino acid sequences are positionally encoded, providing a complete representation of each amino acid and its positional context within the sequence. This step enhances the model’s ability to understand the sequential information and contextual relationships of the amino acid sequences.
- We propose a deep learning architecture that incorporates CRF to better extract sequence features and improve prediction accuracy. The architecture begins by utilizing Transformer Encoders [23] to enhance the feature representation generated during the feature processing stage, aiming to capture richer and more comprehensive sequence information. A hybrid neural network module integrating a convolutional neural network (CNN) [24] architecture and a Bidirectional Long Short-Term Memory Network (BiLSTM) [25] architecture was subsequently developed to capture both long-range and short-range interactions between amino acid residues in a sequence. Finally, the classification probability information for protein secondary structure labels, output by the hybrid neural network module, is fed into the CRF, which leverages its capability to capture interdependencies between secondary structure labels in the sequence, further enhancing prediction accuracy.

## 2. Materials and Methods

### 2.1. Datasets

In order to ensure the data quality and improve the generalization ability of the model, we chose the dataset released in NetSurfP-2.0 [26] as the training data for the model. The dataset provides structural information for a total of 10,792 proteins, including their amino acid sequences and corresponding 3-state and 8-state secondary structure tag sequences. Additionally, five publicly available test datasets were selected to evaluate and compare the performance of protein secondary structure prediction methods: CB513 [27], TS115 [28], NEW364 [18], CASP11 [29], and CASP12 [30]. Among them, CB513 and TS115 are both datasets typically used for protein secondary structure prediction tasks, containing the amino acid sequences of 513 and 115 proteins and their secondary structure information, respectively. CASP11 and CASP12 are competition data provided by the Critical Assessment of Structural Prediction of Proteins (CASP) tournaments. The NEW364 dataset contains 364 proteins with a resolution greater than 2.5 Å and an amino acid sequence length greater than 20. These evaluation datasets have excluded proteins that are duplicates of the training dataset to ensure the objectivity and rigor of the evaluation experiments.

### 2.2. Model Architecture

**Figure 1:**
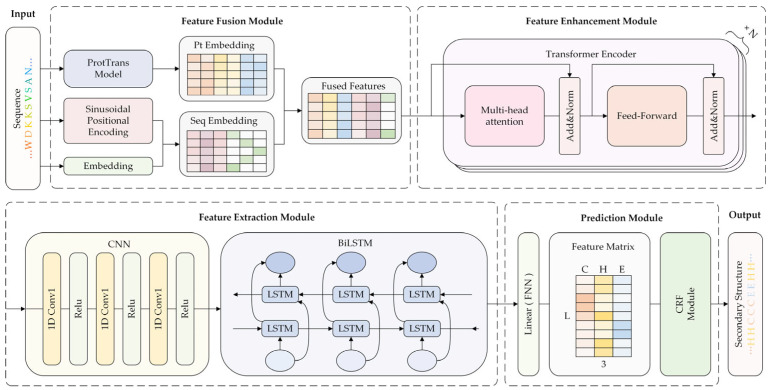
The overall process of ILMCNet.

In this study, we have designed a deep learning network model capable of realizing high-precision prediction of the secondary structure of proteins, which is a network model that integrates a language model and a CRF. To highlight this feature, we combined the initials of the names of the two core modules integrated and defined the name of the model as ILMCNet. ILMCNet is mainly composed of four modules: (1) feature processing module, (2) feature enhancement module, (3) feature extraction module, and (4) classification prediction module. In Figure 1, the overall architecture of the model designed in this paper is demonstrated. In module (1), the protein embeddings generated by the protein language model are fused with the sequence-coded embeddings, and the resulting features will serve as the input to the model. The task of module (2) is to enhance the information representation of the input sequence features by utilizing multiple Transformer Encoders to encode the input features to obtain richer and more comprehensive contextual information. Module (3), which focuses on extracting sequence features, integrates two sub-modules: CNN and BiLSTM. The CNN is designed to extract local features from the amino acid sequence, while the BiLSTM captures long-range dependencies by combining both forward and backward information. In module (4), the output of module (3) first goes to the linear layer to get the secondary structure labeling score for each amino acid residue. Then, the CRF considers the interdependencies between the secondary structures to further find the most probable labeling sequence.

#### 2.2.1. Feature Processing Method

ILMCNet proposes a new feature processing method; on the one hand, PLM is used to generate protein embeddings containing evolutionary information, and on the other hand, the amino acid sequences are feature-encoded to obtain rich amino acid residue information and positional information. Finally, the two are fused to obtain the input feature representation needed in this study.

**Figure 2:**
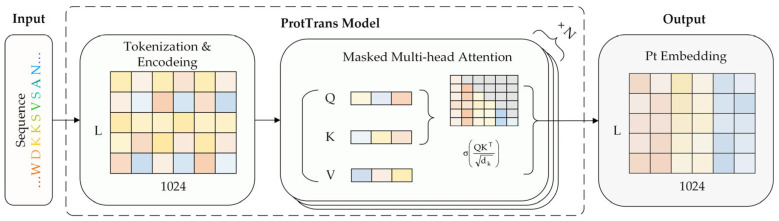
The basic process of protein embedding generation. The ProtTrans model receives the amino acid sequence of the protein (length L, i.e., the number of amino acids in the sequence is L) as an input and generates 1024-dimensional embeddings for each amino acid on this sequence; finally, these L 1024-dimensional amino acid embeddings are concatenated to obtain the protein embedding of the input sequence (L × 1024).

In this study, the ProtTrans model is chosen to generate protein embeddings, and the specific generation process is shown in Figure 2. ProtTrans provides a ProtT5-XL-U50 model, which performs best in each protein downstream task. The model is primarily based on the T5 [31] architecture and contains 24 attention layers with 32 attention heads per layer. Specifically, we chose its half-precision version ProtT5-XL-Half_Uniref50-Enc to generate protein embeddings. Elnaggar et al. has shown that ProtT5-XL-Half_Uniref50-Enc performs comparably to ProtT5-XL-U50.

Additionally, the combination of Token Embedding and Positional Embedding encoding can help the model to understand the meaning of each amino acid in the sequence and its positional information. In order to obtain the position vectors of amino acid sequences, we used Sinusoidal Positional Encoding [32]. This positional coding method essentially calculates positional coding by applying different frequencies of the sine and cosine functions to the position where each amino acid is located in the sequence. The specific calculations for the sine and cosine position codes are as follows:

(1) $$PE_{\left(pos,2i\right)}=sin\left(\frac{pos}{{10,000}^{\frac{2i}{d_{model}}}}\right)$$

(2) $$PE_{\left(pos,2i+1\right)}=\cos\left(\frac{pos}{{10,000}^{\frac{2i}{d_{model}}}}\right)$$

where *pos* represents the position of the amino acid in the sequence, *2i* or *2i + 1* represents a component of the position encoding vector, *2i* represents an even number, and *2i + 1* represents an odd number.

Therefore, position coding using the sine–cosine position coding method can well reflect the position information of each amino acid in the sequence, which in turn helps the model to capture the long-range dependence of the amino acid sequence. Finally, the positional embeddings are further fused with the word embeddings to obtain the sequence-coded embeddings. The input features of ILMCNet are fused from the PLM-generated protein embeddings and the feature representations formed by sequence encoding, which are then transported to the feature enhancement module to await the next step of processing.

#### 2.2.2. Feature Enhancement Module

The fusion features obtained after feature processing of amino acid sequences are input to the Transformer Encoder, and feature enhancement is performed to obtain richer and more comprehensive sequence information. The core of the Transformer Encoder lies in the attention mechanism, and the specific method of calculating the attention is shown in Equations (3) and (4):

(3) $$\left\{\begin{array}{c} Q=h_{t}\cdot W_{Q}\\ K=h_{t}\cdot W_{K}\\ V=h_{t}\cdot W_{V} \end{array}\right.$$

(4) $$Attention(Q,K,V)=Softmax\left(\frac{QK^{T}}{\sqrt{d_{k}}}\right)\cdot V$$

where $h_{t}$ is the input data, *Q* is the query vector, *K* is the key vector, *V* is the value vector, and $W_{Q,K,V}$ is the weight coefficient.

The Transformer Encoder stacks multiple encoder blocks, each of which includes Multi-Head Attention, Feed-Forward Network, and Add and Norm layer. Selection of the optimal number of Transformer layers and the number of Multi-Head Attention heads can be achieved by observing the performance and efficiency of the model under different parameter configurations.

#### 2.2.3. Feature Extraction Module

The feature extraction module of ILMCNet integrates two neural network models, CNN and BiLSTM. Specifically, CNN contains convolutional operations that enable the model to automatically learn and extract feature representations of amino acid sequences. The multilayer convolutional neural network architecture is capable of deeply extracting more complex feature information by stacking multiple one-dimensional convolutional layers. Furthermore, in the BiLSTM architecture, the forward Long Short-Term Memory Network (LSTM) processes the inputs in the natural order of time steps, while the reverse LSTM processes the same inputs in the reverse order. In this way, the final hidden state of each time step is jointly determined by the information from all time steps before and after that step. This integration of bi-directional information flow allows BiLSTM to fully capture the long-range dependence of amino acid sequence features.

#### 2.2.4. Predictive Module

**Figure 3:**
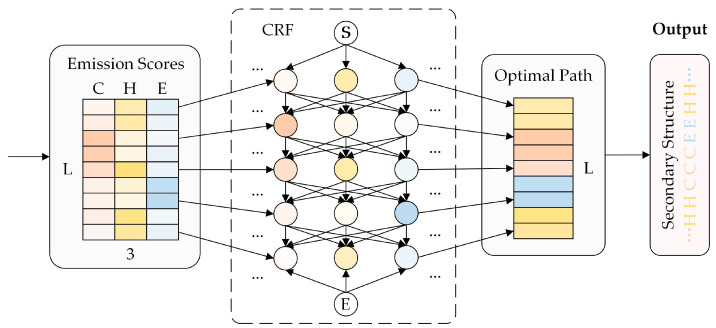
The basic architecture of CRF.

The output of the feature extraction module is first linearly transformed through the fully connected layer (FNN) to align the dimensions of the feature representations obtained with the dimensions of the labels. For example, when performing 3-state secondary structure prediction, the FNN maps the feature vector to a 3-valued distribution probability, also called the firing score (state score), indicating the probability of predicting each amino acid as a 3-state secondary structure label. Subsequently, the firing score is fed into the CRF, which outputs the most probable sequence of predicted secondary structure labels. The basic architecture of the CRF is shown in Figure 3.

One of the two core components of the CRF model is the firing score. The other component is the probability transition matrix between the secondary structure labels, referred to as the transition score. After obtaining these two scores, the probability of a certain amino acid being labeled with a specific secondary structure label can be calculated. The amino acid sequence $X=(x_{1},x_{2},\ldots ,x_{T})$ and the secondary structure labeling sequence $Y=(y_{1},y_{2},\ldots ,y_{T})$ are known, and the computational procedure is demonstrated in Equations (5) and (6):

(5) $$\;Z(X)=\sum_{Y^{\prime }}\;exp\left(\sum_{t=1}^{T}\;\sum_{k}\;\theta_{k}f_{k}(y_{t}^{\prime },y_{t-1}^{\prime },X,t)\right)$$

(6) $$P\left(Y|X\right)=\frac{1}{Z\left(X\right)}\exp\left(\sum_{t=1}^{T}\;\sum_{k}\;\theta_{k}f_{k}\left(y_{t},y_{t-1},X,t\right)\right)$$

where $f_{k}(y_{t},y_{t-1},X,t)$ is the feature function that describes the dependencies between secondary structure labels and between secondary structure labels and amino acid residues. $\theta_{k}$ is the feature weight, which is learned from the training data. Z(X) is the collocation function, which is used for normalization and ensures that the probability of all possible label sequences sums to one.

It is worth noting that when carrying out the calculation of all path scores, if you list all possible paths, then find the firing score and transition score of each path and calculate the scores for summing, this method is undoubtedly inefficient. Viterbi algorithm based on the principle of dynamic programming can effectively solve this problem. Viterbi algorithm calculates the state probability of each time step by recursion and selects the path with the highest cumulative probability at each step, and finally backtracks to get the optimal state sequence of the whole sequence. This algorithmic idea can greatly improve the efficiency of the summation calculation. Equation (7) represents the computational idea of Viterbi algorithm:

(7) $$\delta_{t}(y_{t})=\underset{y_{t-1}}{max}\;\left[\delta_{t-1}(y_{t-1})+\theta_{k}f_{k}(y_{t},y_{t-1},X,t)\right]$$

where $\delta_{t}(y_{t})$ denotes the optimal cumulative score when the label sequence has $y_{t}$ as the tth label from the starting position of the sequence until time step t. After the optimal path score $\delta_{T}(y_{T})$ is computed at time step t, the optimal labeling sequence $Y^{*}$ for the whole sequence is found by backtracking. Based on the above processing, the model can find out the maximum possible predicted secondary structure labeling sequence of the target amino acid sequence.

### 2.3. Loss Functions

In the prediction process, after obtaining a reasonable labeling probability transition matrix, it is only necessary to combine it with the Emission Score to select the path with the maximum probability using the Viterbi algorithm, and this process constitutes the decoding stage. During training, the log-likelihood function is maximized to measure the difference between the sequence labeling predicted by the model and the actual labeling. Since, in deep learning, model parameters are generally optimized by minimizing the loss function, the model uses the negative log-likelihood function as the loss function. The negative log-likelihood function is calculated in Equations (8) and (9):

(8) $$\;P\left(Y|X,\theta \right)=\frac{1}{Z\left(X,\theta \right)}\exp\left(\sum_{i,k}\;\theta_{i,k}f_{k}\left(y_{i-1},y_{i},X\right)\right)$$

(9) $$L_{NLL}=-\log P\left(Y|X\right)$$

where $f_{k}(y_{i-1},y_{i},X)$ denotes the eigenfunction, *k* denotes the index of the eigenfunction, $y_{i-1}$ and $y_{i}$ denote the labeling of the i-1st and i-th position in the sequence, respectively, θ is used to calculate the weight of the eigenfunction, and Z(X,θ) is the normalization factor (the collocation function), which is used to ensure that the total probability of all the possible labeled sequences is 1. Minimizing the loss function using gradient descent or other optimization algorithms, this approach better captures the interdependencies between secondary structure labels, thereby improving prediction accuracy.

## 3. Results

### 3.1. Experimental Setup

#### 3.1.1. Assessment of Indicators

The performance evaluation of the ILMCNet model primarily relies on two key metrics. One of these metrics is the calculation of the percentage of secondary structure classes corresponding to amino acid residues in a sequence that is accurately predicted, known as precision, which is described in Equation (10):

(10) $$Q_{m}=\frac{\sum_{i=1}^{m}A_{i}}{N}$$

where $Q_{m}$ represents the accuracy, m represents the number of secondary structure categories, *N* represents the total number of residues in the target amino acid sequence, and $A_{i}$ represents the number of amino acids correctly predicted for the secondary structure category.

Another evaluation metric is fragment overlap (Sov), which not only focuses on the percentage of individual residue positions predicted correctly but also emphasizes the consecutive overlapping fragments between the actual structure and the predicted structure. The specific calculation of the Sov score is shown in Equation `:

(11) $$\mathrm{Sov}=\frac{100\times \sum_{S_{0}}\;\left[\frac{minov(S_{1},S_{2})+\sigma (S_{1},S_{2})}{maxov(S_{1},S_{2})}\cdot length(S_{1})\right]}{N}$$

where *N* represents the total number of amino acid residues in the sequence, $S_{1}$ is the true fragment, $S_{2}$ is the predicted fragment, $S_{0}$ represents the fragment with the same structure in both $S_{1}$ and $S_{2}$, $maxov\left(S_{1},S_{2}\right)$ represents the longest overlapping fragment in both $S_{1}$ and $S_{2}$, $minov\left(S_{1},S_{2}\right)$ represents the shortest overlapping fragment in both $S_{1}$ and $S_{2}$, and $\sigma \left(S_{1},S_{2}\right)$ represents the similarity score between $S_{1}$ and $S_{2}$. Equation (12) shows the computation of $\sigma \left(S_{1},S_{2}\right)$:

(12) $$\sigma \left(S_{1},S_{2}\right)=min\left\{\begin{array}{c} \;\;\;\;\;maxov\left(S_{1},S_{2}\right)-minov\left(S_{1},S_{2}\right)\\ minov\left(S_{1},S_{2}\right)\\ int\left[len\left(S_{1}\right)\right]/2\\ int\left[len\left(S_{2}\right)\right]/2 \end{array}\right.$$

#### 3.1.2. Experimental Environment

Hardware equipment used in this study:

- CPU: Intel Xeon Gold 5218R, 2.10 GHz (Intel, Santa Clara, CA, USA).
- GPU: RTX 2080Ti (11 GB), cuda11.1 (Nvidia, Santa Clara, CA, USA).
- Memory: 32 GB.

This study is based on the Python 3.7 environment and uses PyTorch framework to implement the model and complete the experiments. Based on the above experimental environment, we fix the parameters and size of ILMCNet and set the training epochs to 100; then, it takes about 10 h to train the optimal model.

### 3.2. Performance Evaluation

To evaluate the performance of the ILMCNet model, we compared it with two types of methods separately. The first class of methods includes DeepProtVec [12], DeepSeqVec, ESM-1b, and NetSurfP-3.0, all of which use amino acid sequences as unique inputs, and were compared and evaluated on the CASP12, NEW364, TS115, and CB513 test sets, respectively. The second class of methods are identical in that they all incorporate other input features such as PSSM. ILMCNet was compared with the second class of methods on the CASP11, CASP12, and CB513 test sets: SSPro, Porter 5, MUFOLD-SS, and DNSS2, respectively.

Table 1 presents the comparison of the ILMCNet model with the first category of methods for the prediction of 3-state and 8-state secondary structures across the four test sets. Table 2 illustrates the results of the comparison between the ILMCNet model and the second category of methods on three test sets. It is evident that the ILMCNet model not only outperforms the single sequence input model on every test set, but also shows a clear advantage over methods that incorporate additional information in the input features.

In addition, we specifically compare the performance of ILMCNet with NetSurfP-3.0, NetSurfP-2.0, SPOT-1D-Single [33], and SPOT-1D-LM [34] on the CASP14_FM dataset [35]. The specific comparison results are shown in Table 3. From the experimental results, it can be seen that ILMCNet performs very well on the CASP14_FM dataset.

The strong performance of ILMCNet in the task of protein secondary structure prediction is due to its unique structural design. The feature processing approach designed by ILMCNet successfully extracts rich and comprehensive feature information from amino acid sequences alone. Additionally, the model integrates three deep learning architectures to create a hybrid framework, allowing for more thorough and effective utilization of the feature information. In the prediction stage, the incorporation of CRF emphasizes the dependencies between secondary structure labels, significantly enhancing the model’s predictive performance.

### 3.3. Parametric Analysis

For the hyperparametric experiments, we selected the CB513 dataset as a test set to explore the performance of the model in the secondary structure prediction task. The CB513 dataset is one of the most used datasets in protein secondary structure prediction studies and has been used as benchmark data by most of the similar studies.

#### 3.3.1. Sequence-Encoded Feature Size

The size of input features from encoding amino acid sequences using word and position embeddings significantly impacts model performance. Larger feature dimensions offer more detailed positional information, but also increasing computational demands and overfitting risks. Conversely, smaller dimensions may result in insufficient information. Specifically, experimental results show that the model performs best when the feature size is 512 (Supplementary Figure S1).

#### 3.3.2. Parameter Analysis of the Transformer Module

Selecting the optimal number of Transformer layers and attention heads is crucial for model performance. Too few layers hinder feature representation learning, while too many increase complexity and slow execution. Results show that as H increases, model performance improves steadily (Supplementary Figure S2), and when L = 6 and H = 8, the performance of the model is relatively optimal, and the model is more sensitive to the change of H compared to L.

#### 3.3.3. Number of Convolutional Layers

In a multilayer convolutional module, increasing the number of convolutional layers enhances the model’s ability to learn deeper feature representations. However, this also raises computational complexity and training costs. By varying the number of convolutional layers, we can assess the impact on model performance. Experimental results show that ILMCNet performs best when using three convolutional layers (Supplementary Figure S3).

#### 3.3.4. Number of Hidden Units

Increasing the number of hidden units in BiLSTM enhances the learning ability of the ILMCNet model, allowing it to capture more complex amino acid characterization information. However, too many hidden units can lead to increased model parameters, higher computational costs, and potential overfitting. In our hyperparameter experiments, we adjusted the number of hidden units to find an optimal balance between performance and efficiency. The experimental results show that the model performance improves with the increase of hidden units when the hidden units are set to 32, 64, 128, and 256, peaking at 256 (Supplementary Figure S3).

### 3.4. Ablation Experiment

The ablation experiments in this study all used the CB513 dataset as the evaluation test set, with accuracy and Sov scores as the measures.

#### 3.4.1. Data Ablation

To explore the effect of different amino acid sequence coding methods on the ILMCNet model, we performed ablation experiments concerning the coding methods (Supplementary Table S1).

Overall, the absence of either protein embedding or sequence embedding led to a noticeable decline in model performance, while their combined use achieved the best results. Notably, protein embedding contributed more significantly, enhancing accuracy by 20.56% in the 8-state secondary structure prediction task. This improvement is largely due to the protein language model’s strong learning capability, allowing it to capture evolutionary information without needing additional features. In addition, positional encoding using the sine–cosine method enhances the model’s understanding of each amino acid’s position in the sequence, improving the recognition of dependencies among them.

#### 3.4.2. Model Ablation

To explore the impact of each module on model performance, we conducted ablation experiments by sequentially removing each module from the ILMCNet model, while keeping the coding method and training parameters unchanged (Supplementary Table S2).

According to experimental results, the introduction of different network modules produces different levels of improvement in model performance. It is clear that the CRF has the most significant impact on model performance. This is attributed to the fact that CRF helps the model capture the interdependence between secondary structure labels in a sequence. Meanwhile, this result confirms the centrality of CRF in this study. Based on the above analysis, it can be concluded that all the network modules included in ILMCNet contribute to protein secondary structure prediction; especially, CRF performs better.

### 3.5. Visualization Comparison

PyMOL [36], as a tool that can map secondary structures to their corresponding tertiary conformations, is able to visualize how the ILMCNet model compares to existing techniques [37]. The AlphaFold series, as the best-performing method in the field of structure prediction, deserves extra attention and analysis for the results of the comparisons with it. We randomly selected two proteins (PDB IDs 7GSU and 7FPL, respectively) from the PDB [38] that were released after 12 January 2023 (the cutoff date for selection of the AlphaFold 3 training set), and then used PyMOL 3.0 to compare them by two methods.

**Figure 4:**
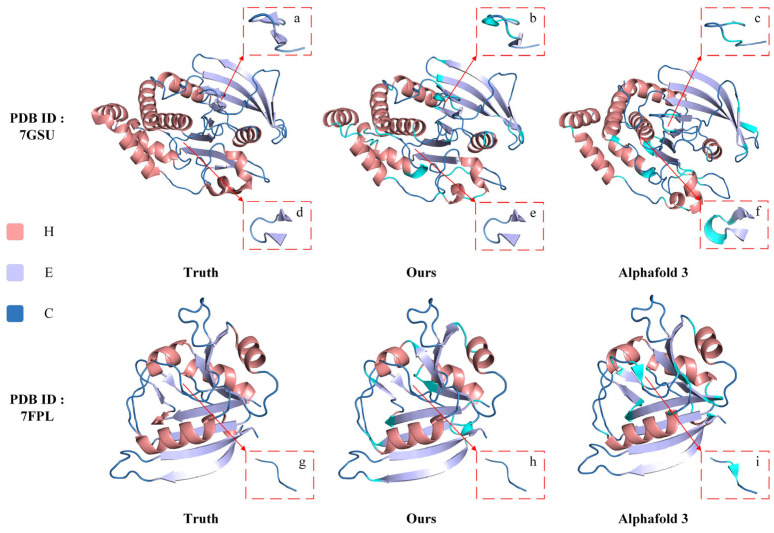
The visualization results of the first comparison method. For the protein with PDB ID 7GSU, local enlargements (**a**–**c**) show the structural details on the amino acid fragments at positions numbered 112–122; local enlargements (**d**–**f**) show the structural details on the amino acid fragments at positions numbered 73–80, respectively. Similarly, for the protein with PDB ID 7FPL, local enlargements (**g**–**i**) show the structural details on the amino acid fragment at position number 51–56, respectively.

The visualization comparison results for the first method are shown in Figure 4. PyMOL generates a real structure image of the protein using the structure information file from the PDB, which includes both 3D structural data and the corresponding secondary structure information for each amino acid. We modified the secondary structure information in the PDB file based on the sequence predicted by ILMCNet to create the corresponding 3D structure display in PyMOL. Additionally, AlphaFold 3 offers a publicly accessible structure prediction tool that can predict protein 3D structures online and produce a result file recognized by PyMOL. To clearly highlight the differences between the predicted and actual structures from ILMCNet and AlphaFold 3, we used cyan for distinction. In particular, the local zoomed-in images show the structural details on different amino acid fragments.

**Figure 5:**
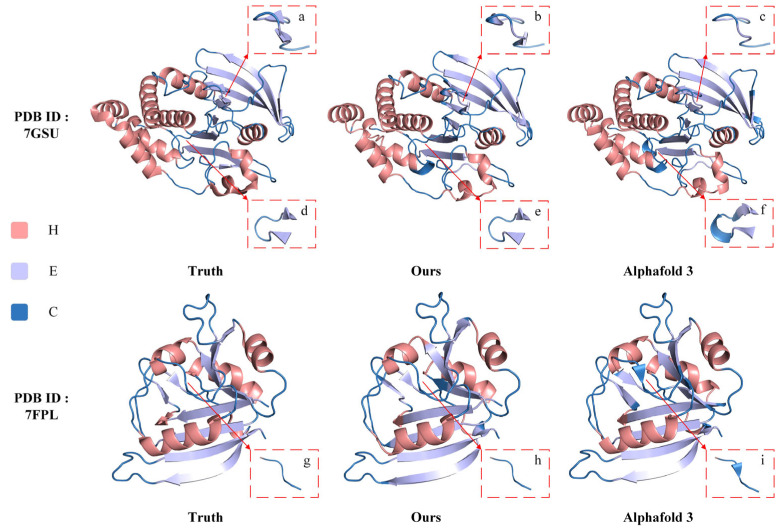
The visualization results of the second comparison method are presented. As labeled in Figure 4, local enlargements (**a**–**i**) highlight the structural details of the three conformations across different amino acid fragments.

In this second method, we do not directly use the result file from AlphaFold 3. Instead, we first employ the VADAR to extract the corresponding secondary structure sequences from the AlphaFold result file. We then modify the PDB file accordingly and use PyMOL to visualize the changes. This approach allows us to focus solely on the differences between the secondary structures predicted by ILMCNet and AlphaFold 3, without considering 3D structural variability. In Figure 5, the variability of the predictions compared to the real structure is indicated by mismatches in secondary structure types and their corresponding colors. For instance, if a helical segment is colored sky blue, it suggests that the actual secondary structure should be helical, however, it was incorrectly predicted as another type.

The comparative results in Figure 4 and Figure 5 show that AlphaFold 3 generally predicts better outcomes and outperforms ILMCNet for most fragments. However, AlphaFold 3 exhibits instability in predicting the number of fragments. Specifically, for the protein 7GSU, AlphaFold 3 predicted two fewer folded fragments compared to the true results and ILMCNet for the amino acid fragments at positions 112–122, while it predicted one additional helical fragment compared to the true results and ILMCNet for the amino acid fragments at positions 73–80. For the protein 7FPL, AlphaFold 3 predicted one extra folded fragment at the amino acid fragments numbered 51–56. Although the results predicted by ILMCNet did not exactly match the actual structures, the total number of predicted protein secondary structure fragments is approximately equal to the actual results.

## 4. Discussion

The ILMCNet model proposed in this study offers significant advantages for protein secondary structure prediction using a single amino acid sequence as input. Firstly, the model leverages a protein language model, replacing traditional evolutionary information features like PSSM with protein embeddings, and combines word embeddings and positional embeddings to create a rich representation of protein features. Secondly, in designing the prediction model, we not only aimed to construct a high-performance neural network but also incorporated a CRF to derive optimal secondary structure tag sequences. This approach enhances the model’s ability to understand the dependencies between secondary structure tags in amino acid sequences, thereby improving prediction performance. A series of experiments in this paper demonstrate that ILMCNet performs excellently in both 3-state and 8-state secondary structure prediction tasks. The results validate that our proposed feature processing and the integration of CRF significantly enhance protein secondary structure prediction. Additionally, visual comparisons with AlphaFold 3 show that, although ILMCNet may be slightly behind in overall prediction results, its accuracy in predicting the number of fragments surpasses that of AlphaFold 3. Moreover, ILMCNet will perform better in the field of protein secondary structure prediction if we can conduct deeper research based on the following two key points: first, finding or training a protein language model that is more suitable for the task of protein secondary structure prediction so that the protein embeddings it generates contain more comprehensive evolutionary information; and second, exploring the improvement of CRFs or their integration in order to fully utilize its role in protein secondary structure prediction tasks. For example, by combining more biological knowledge or analyzing the biological characteristics of protein secondary structure, we can find the best CRF constraint rules to improve the prediction accuracy of secondary structure tagged sequences.

## Figures and Tables

**Table 1:** A comparison of results of the ILMCNet model with the first class of methods on four test sets.

Model	CASP12		NEW364		CB513		TS115	
	${Acc}_{3}$	${Acc}_{8}$	${Acc}_{3}$	${Acc}_{8}$	${Acc}_{3}$	${Acc}_{8}$	${Acc}_{3}$	${Acc}_{8}$
DeepProtVec	0.6290	0.4970	0.6470	0.5330	0.6370	0.4890	0.6650	0.5440
DeepSeqVec	0.7302	0.6103	0.7600	0.6484	0.7701	0.6275	0.7901	0.6720
ESM-1b	0.7689	0.6604	0.8263	0.7130	0.8392	0.7023	0.8480	0.7344
NetSurfP-3.0	0.7780	0.6644	0.8330	0.7290	0.8503	0.7204	0.8590	0.7572
Ours	0.8203	0.6990	0.8451	0.7417	0.8639	0.7510	0.8672	0.7759

**Table 2:** A comparison of results of the ILMCNet model with the second class of methods on three test sets.

Model	CASP11		CASP12		CB513	
	${Acc}_{3}$	${Acc}_{8}$	${Acc}_{3}$	${Acc}_{8}$	${Acc}_{3}$	${Acc}_{8}$
SSPro5.2	0.7772	0.6687	0.7616	0.6468	0.7764	0.659
Porter 5	0.8316	0.7015	0.8058	0.6848	0.8381	0.6941
MUFOLD-SS	0.8128	0.6905	0.7948	0.6665	0.8171	0.6819
DNSS2	0.8284	0.7304	0.8095	0.7082	0.8256	0.7336
Ours	0.8533	0.7556	0.8203	0.7130	0.8639	0.7510

**Table 3:** A comparison of results between ILMCNet and other methods on the CASP14_FM dataset.

Model	${Acc}_{3}$	${Acc}_{8}$
NetSurfP-3.0	0.6010	0.6070
NetSurfP-2.0	0.5810	0.6180
SPOT-1D-Single	0.5760	0.5720
SPOT-1D-LM	0.6150	0.6230
Ours	0.7552	0.6930

## Data Availability

We established a webserver to implement the proposed method, which is currently accessible via https://bioinfor.nefu.edu.cn/ILMCNet/ (accessed on 21 July 2024). Moreover, the source code and dataset of ILMCNet have been uploaded to https://github.com/Seowhi/ILMCNet/tree/master (accessed on 21 July 2024).

## Supplementary Materials

The following supporting information can be downloaded at: https://www.mdpi.com/article/10.3390/genes15101350/s1. Figure S1: effect of sequence feature encoding size on model performance; (A) accuracy and Sov scores corresponding to dividing the secondary structure representation into three categories; (B) accuracy and Sov scores corresponding to dividing the secondary structure representation into eight categories. Figure S2: to explore the effects of Transformer layer L and multi-note head H on model performance; (A) when the secondary structure representation is divided into three categories, L and H are set to the accuracy corresponding to different sizes; (B) when the secondary structure notation is divided into three categories, L and H are set to the Sov scores corresponding to different sizes; (C) when the secondary structure representation is divided into eight categories, L and H are set to the accuracy corresponding to different sizes; (D) when the secondary structure representation is divided into eight categories, L and H are set to Sov scores corresponding to different sizes. Figure S3: exploring experimental results on optimal hyperparameter settings; (A) optimal number of convolutional layers included in a multilayer convolutional module; (B) optimal hidden cell count. Table S1: experimental results using different coding methods. Table S2: experimental results with the introduction of different modules.
