# Supplementary material for: ILMCNet: A Deep Neural Network Model That Uses PLM to Process Features and Employs CRF to Predict Protein Secondary Structure
Source: Genes (Basel). 2024 Oct 21;15(10):1350. doi: 10.3390/genes15101350 (PMC11507629; doi:10.3390/genes15101350)
Supplement: Supplementary file 1 [file genes-15-01350-s001.zip › genes-3222786-supplementary.pdf]

Supplementary Material

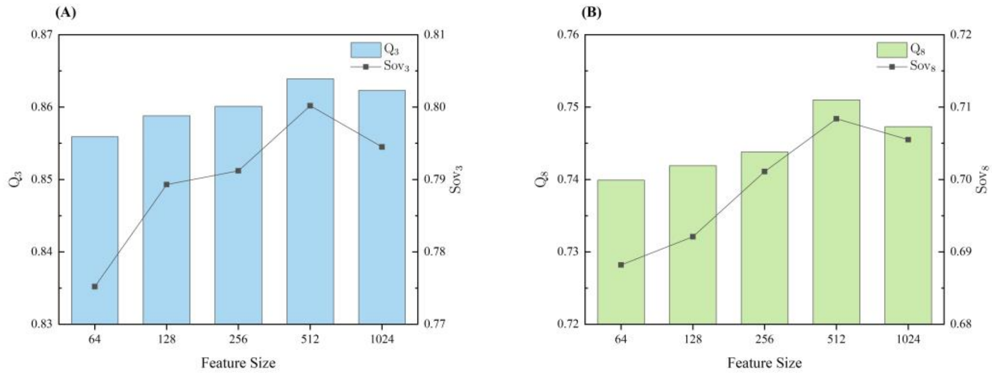

Figure S1. Effect of sequence feature encoding size on model performance: (A) Accuracy and Sov scores corresponding to dividing the secondary structure representation into three categories; (B) Accuracy and Sov scores corresponding to dividing the secondary structure representation into eight categories.

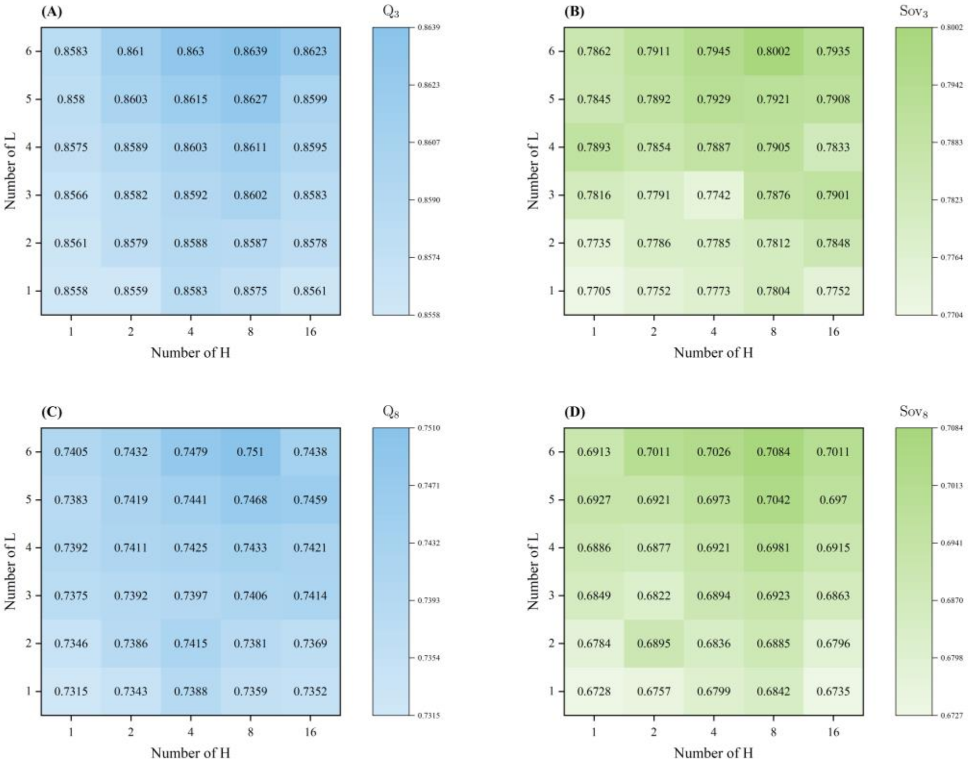

Figure S2. To explore the effects of transformer layer L and multi-note head H on model performance: (A) when the secondary structure representation is divided into three categories, L and H are set to the accuracy corresponding to different sizes; (B) When the secondary structure notation is divided into three categories, L and H are set to the Sov scores corresponding to different sizes; (C) When the secondary structure representation is divided into eight categories, L and H are set to the accuracy corresponding to different sizes; (D) When the secondary structure representation is divided into eight categories, L and H are set to Sov scores corresponding to different sizes.

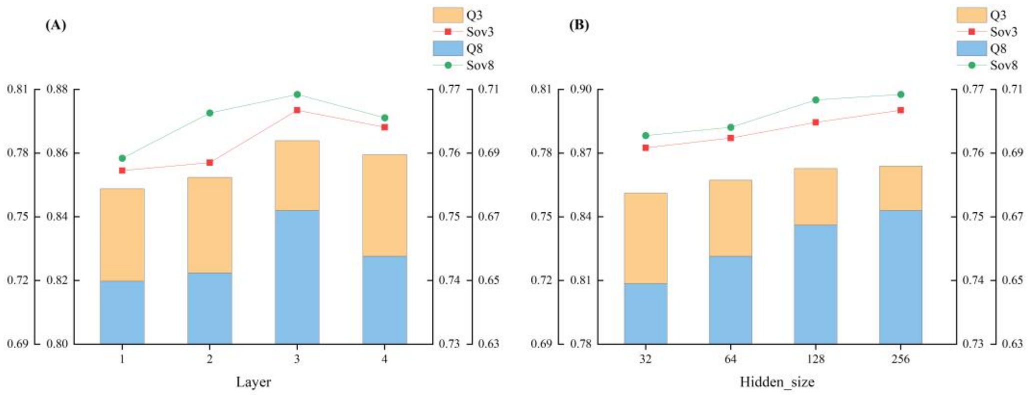

Figure S3. Exploring experimental results on optimal hyperparameter settings: (A) Optimal number of convolutional layers included in a multilayer convolutional module; (B) Optimal hidden cell count.

Table S1. Experimental results using different coding methods.

Data	Acc_3	Sov_3	Acc_8	Sov_8
No Pt_embedding	0.6949	0.5956	0.5454	0.4818
No Seq_ embedding	0.8584	0.7922	0.7421	0.7046
**Full**	**0.8639**	**0.8002**	**0.7510**	**0.7084**

Table S2. Experimental results with the introduction of different modules.

Network	Acc_3	Sov_3	Acc_8	Sov_8
No Transformer Encoder	0.8569	0.7761	0.7429	0.6946
No CNN	0.8502	0.7697	0.7394	0.6918
No BiLSTM	0.8537	0.7715	0.7406	0.6925
No CRF	0.8478	0.7608	0.7339	0.6884
**Full**	**0.8639**	**0.8002**	**0.7510**	**0.7084**
